# Is the Hallux Interphalangeal Ossicle Clinically Relevant? A Cross-Sectional Study on Its Prevalence and Biomechanical Implications

**DOI:** 10.3390/life16050816

**Published:** 2026-05-14

**Authors:** Ana Isabel Marcos, Salomón Benhamú-Benhamú, Antonio Córdoba-Fernández

**Affiliations:** 1 Departament of Podiatry, Universidad de Sevilla, 41009 Seville, Spain; podologamarcoscasado@gmail.com (A.I.M.); benhamu@us.es (S.B.-B.); 2 Departament of Podiatry, Liderazgo DS-30 Bases Biomédicas del pie que Afectan al Apoyo y la Marcha, Instituto de Biomedicina de Sevilla, IBiS/Universidad de Sevilla, 41009 Sevilla, Spain

**Keywords:** accessory bone, foot, hallux, interphalangeal joint, hallucal interphalangeal ossicle

## Abstract

Background: The hallux interphalangeal ossicle (HIO) is commonly considered as an incidental anatomical variant; however, its biomechanical role remains poorly understood. This study aimed to investigate the influence of HIO on hallux joint biomechanics. Methods: A cross-sectional correlational study was conducted, including 419 feet (218 individuals). The presence of HIO was assessed using ultrasound imaging. Range of motion (ROM) of the metatarsophalangeal joint (MTPJ) and interphalangeal joint (IPJ) were evaluated under both open kinetic chain and dynamic conditions. Statistical comparisons between HIO and non-HIO groups were made, and receiver operating characteristic (ROC) curve analysis was used to assess the discriminative capacity of IPJ ROM. Results: HIO was present in 48% of cases and was bilateral in all participants. Individuals with HIO exhibited significantly greater IPJ extension under both open kinetic chain and dynamic conditions (*p* < 0.05). No significant differences were observed in MTPJ ROM between groups. A positive, albeit variable, relationship was found between ossicle size and IPJ extension. ROC analysis demonstrated moderate discriminative ability of IPJ ROM for detecting HIO (sensitivity 63.2%, specificity 54.6%). Conclusions: The presence of HIO is associated with increased IPJ extension, suggesting a measurable influence on hallux biomechanics. These findings support the notion that the HIO is a biomechanically relevant structure rather than purely incidental an asymptomatic anatomical variant. Increased IPJ extension may represent an early functional adaptation with potential clinical implications.

## 1. Introduction

The prevalence and anatomical distribution of sesamoid and accessory bones in the foot have been widely reported, although considerable variability exists across populations and methodologies [1]. Numerous supernumerary bones have been identified in the forefoot, most of which are asymptomatic and are typically detected incidentally during imaging studies [2].

The os interphalangeus, also known as the hallucal interphalangeal ossicle (HIO), is an accessory structure located at the interphalangeal joint (IPJ) of the hallux. Its reported prevalence varies widely in the literature, partly due to differences in detection methods and the degree of ossification [1]. Radiographic studies are limited to identifying fully ossified forms, whereas cadaveric investigations suggest a substantially higher prevalence [3,4]. In this context, ultrasound (US) has emerged as a sensitive imaging modality capable of detecting the HIO even in non-ossified forms [5].

Although previously described as a sesamoid bone within the flexor hallucis longus (FHL) tendon, current anatomical evidence indicates that the HIO is embedded within the joint capsule and separated from the tendon by a bursal structure. In the absence of the HIO, this bursa is not present, and the tendon inserts directly into the capsule [3,4,5]. These anatomical relationships suggest a potential role in modulating tendon mechanics and joint function.

From a functional perspective, the plantar location of the HIO has been associated with several anatomical and biomechanical alterations, as well as clinical conditions [6,7,8,9]. The so-called os interphalangeus syndrome has been described as a clinical entity characterized by IPJ hyperextension, painful plantar hyperkeratosis, and hallux limitus (HL), highlighting the potential clinical relevance of this structure. The relationship between IPJ hyperextension and HL is complex and likely bidirectional. The HIO is strategically positioned to promote early dorsiflexion of the proximal phalanx and plantar subluxation during propulsion. This mechanism may contribute to HL, with compensatory hyperextension occurring at the IPJ. Conversely, increased IPJ dorsiflexion has been observed in individuals with HL, and the presence of HIO has been associated with a higher incidence of postoperative IPJ complications following first MTPJ arthrodesis [10,11].

Despite these observations, the biomechanical role of the HIO and its influence on the functional interplay between the IPJ and MTPJ remain insufficiently understood. Therefore, the aim of this study was to investigate the biomechanical implications of the HIO on hallux function. We hypothesized that the presence of HIO alters the functional coupling between the IPJ and MTPJ and modulates FHL tendon mechanics, thereby affecting joint function.

## 2. Materials and Methods

### 2.1. Study Design and Participants

A cross-sectional correlational study was conducted in the Clinical Podiatry Area at the University of Seville. Participants were recruited through convenience sampling among undergraduate students enrolled in the Podiatry Degree program.

All participants provided written informed consent prior to inclusion and participated voluntarily without financial compensation. The study adhered to the principles of the Declaration of Helsinki and was approved by the Research Ethics Committee of the Virgen Macarena and Virgen del Rocío University Hospitals (Approval Code: 0321-N-21). Participant confidentiality was ensured anonymized numerical coding.

### 2.2. Sample Size Calculation

Sample size estimation was based on the reported HIO prevalence. An a priori power analysis was performed using G*Power software (version 3.1.9; Universität Kiel, Kiel, Germany) for the comparison of two independent means (one-tailed). Assuming a medium effect size (d = 0.5), α = 0.05, and power (1 − β) = 0.80, resulting in a minimum of 189 feet per group.

The unit of analysis was the foot, as right and left feet may present different clinical characteristics within the same individual. A total of 419 feet were included: 201 classified as HIO-positive and 218 as HIO-negative.

### 2.3. Eligibility Criteria

Participants aged 18–40 years were eligible. Inclusion criteria required absence of prior trauma, foot surgery, fractures, or rheumatic disease affecting joint mobility. Exclusion criteria for both groups included structural HL (≤55° dorsiflexion), neuromuscular disorders, generalized joint laxity, or any condition affecting first ray or hallux mobility.

### 2.4. Ultrasound Assessment

Ultrasound examination Ultrasound examinations were performed to determine the presence of HIO using a standardized protocol in longitudinal and transverse planes. The most clearly defined image of the ossicle was selected for measurement. All assessments were performed using a high-frequency linear transductor (ALPINION, Seoul, Republic of Korea). A consistent amount of US gel was applied maintaining minimal probe pressure to avoid tissue deformation. All examinations were conducted by a single experienced examiner to ensure consistency.

### 2.5. Clinical and Kinematic Assessment

Following demographic data collection, participants were positioned supine on an examination table. Bilateral assessment of the hallux included extension at the MTPJ, extension at the IPJ, and maximum flexion at the IPJ. Measurements were obtained in an open kinetic chain under standardized conditions: knee extended, subtalar joint in neutral position, and ankle positioned at 90°. Images were captured from a medial view using anatomical landmarks as reference points. Additionally, dynamic gait analysis during the propulsion phase was conducted using Kinovea software (version 0.9.5). Each measurement was performed twice by the same examiner, and the mean value was used for statistical analysis. To minimize the risk of bias, a standardized participant positioning and measurement protocol was implemented, and the same calibrated equipment was used throughout the study. The assessment was performed by a single examiner to reduce inter-observer variability, and the examiner was blinded (HIO to status) during angle measurements.

To assess intra-observer reliability, a subset of measurements (15% of the sample) was re-evaluated after a two-week interval under identical conditions. Reliability was quantified using the intraclass correlation coefficient (ICC).

### 2.6. Statistical Analysis

Statistical analyses were conducted using SPSS software (version 27.0 for Windows; SPSS Inc., Chicago, IL, USA). Quantitative variables were expressed as mean ± standard deviation (SD) or median and interquartile range (IQR), depending on data distribution. Categorical variables were expressed as frequencies and percentages. Normality was assessed using Kolmogorov–Smirnov test. Between-group comparisons (HIO vs. non-HIO) were performed using the independent samples *t*-test or Mann–Whitney U test, as appropriate. The chi-square test was used for categorical variables such as sex. Receiver operating characteristic (ROC) curve analysis was used to evaluate the discriminative capacity of IPJ range of motion for the presence of HIO, including calculation of the area under the curve (AUC), optimal cut-off points, and associated sensitivity and specificity. Binary logistic regression analysis was conducted to assess the independent association between joint range of motion and HIO presence. All the comparisons were considered significant at *p* < 0.05.

## 3. Results

The intraclass correlation coefficients for the variables measured on the first MPJ and hallux IPJ were first MPJ dorsiflexion 0.993 (0.995–0.997; 95% CI) and hallux IPJ extension 0.963 (0.883–0.994; 95% CI).

The sample comprised 419 feet of 76 men and 142 women, of whom 48.0% (n = 201) presented an interphalangeal ossicle. No statistically significant differences were observed between groups regarding sex (*p* = 0.581). In all cases within the HIO group, the ossicle was present bilaterally. Overall, 66.1% of participants reported regular engagement in sports activities, with soccer, CrossFit, and running being the most frequently practiced.

No statistically significant differences were observed between the HIO and non-HIO groups in terms of anthropometric variables, including weight, height, and body mass index (Table 1).

Morphologically, the HIO was visualized in the US as a hyperechoic structure with posterior acoustic shadowing, located deep to the interphalangeal joint and separate from the flexor hallucis longus tendon. In participants with HIO, the mean ossicle size was 14.4 ± 4.8 mm × 31.0 ± 10.0 mm in the longitudinal axis and 18.0 ± 14.7 mm × 46.2 ± 16.4 mm in the transverse axis (Table 2).

For the overall sample, the mean maximum ROM of the MTPJ in an open kinetic chain was 69.3° ± 14.8, with a median of 70° and an interquartile range (IQR) of 60.0–79.1°. Under dynamic conditions, the mean MTPJ ROM decreased to 44.4° ± 12.4 (median: 45°; IQR: 36.3–52.0°), reflecting the expected functional reduction during gait. No statistically significant differences were found between the HIO and non-HIO groups in MTPJ ROM, either in open kinetic chain or during dynamic assessment (Table 2).

Regarding the IPJ, the mean maximum ROM in an open kinetic chain for the total sample was 18.4° ± 11.3 (median: 18°; IQR: 10–26°). During dynamic conditions, the mean ROM was 15.2° ± 11.9 (median: 15.1°; IQR: 8.3–21.3°), with considerable variability observed across participants. Mean IPJ flexion ROM under non-weight-bearing conditions was 57.2° ± 12.9 (median: 58°; IQR: 50.0–65.9°), indicating a relatively homogeneous distribution within the sample. Statistical analysis revealed significant differences in IPJ extension between groups. Participants with HIO exhibited significantly greater IPJ extension compared to those without HIO, both in open kinetic chain and during dynamic assessment. These findings indicate that the effect of HIO on IPJ mobility is consistent under both static and functional conditions. In contrast, no significant differences were observed between groups in IPJ flexion ROM measured under non-weight-bearing conditions (Table 3 and Table 4).

**Figure 1:**
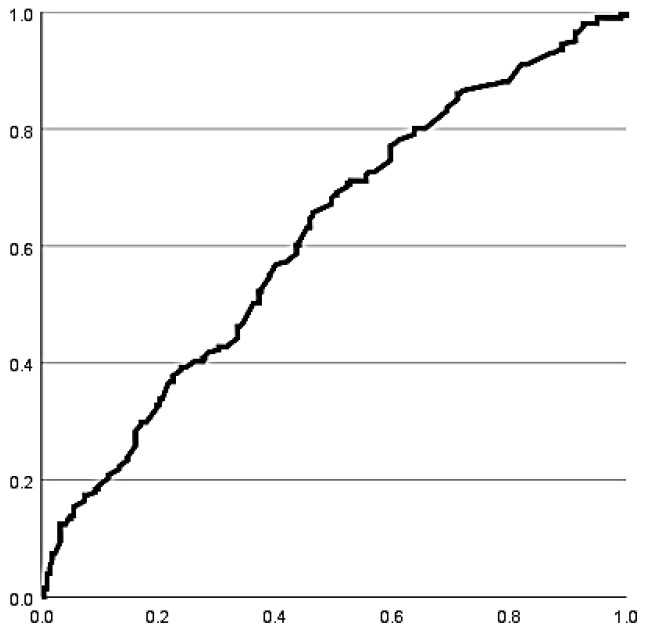
ROC curve for the ROM in IPJ extension on download.

Receiver operating characteristic (ROC) curve analysis demonstrated a moderate ability of IPJ extension ROM to discriminate between the presence and absence of HIO. Sensitivity was 63.2% (95% CI: 56.3–69.5%) and specificity was 54.6% (95% CI: 48.0–61.1%) (Figure 1), indicating limited diagnostic performance.

**Figure 2:**
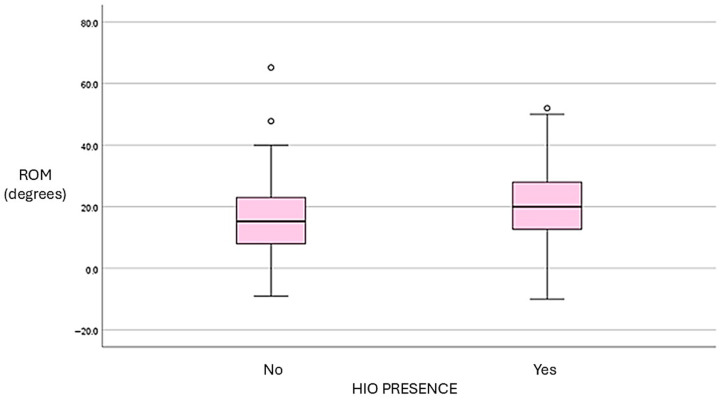
Range of motion in extension at the level of the IPJ in open kinetic chain.

**Figure 3:**
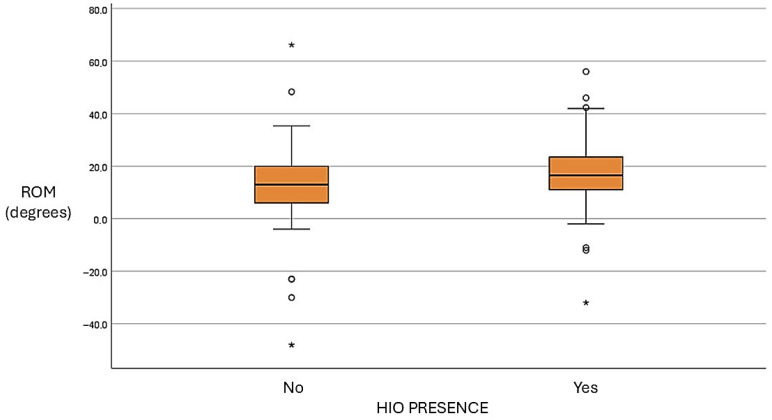
Range of motion in extension at the level of the IPJ in dynamic conditions.

A positive trend was observed between HIO size and IPJ extension ROM, particularly in open kinetic chain conditions. Participants with larger ossicles tended to exhibit greater extension. However, this relationship was not strictly linear, and substantial dispersion of values suggests considerable interindividual variability (Figure 2 and Figure 3).

A logistic regression analysis was performed to evaluate the association between hallux IPJ extension and the presence of an interphalangeal ossicle. In open kinetic chain, range of motion in extension at the level of the IPJ showed a significant positive association with the presence of an interphalangeal ossicle. Specifically, each one-degree increase in extension was associated with a 4% increase in the odds of presenting an ossicle (OR = 1.04; 95% CI: 1.02–1.06; *p* < 0.001). Regarding IPJ extension dynamics, although higher mean values were observed in the group with ossicle, this variable did not reach statistical significance (*p* = 0.141).

## 4. Discussion

The prevalence of the HIO reported in the literature shows considerable variability, largely depending on the imaging modality used and the characteristics of the studied population. Meta-analyses based on radiographic studies have estimated a pooled prevalence of approximately 21.1% [1], whereas computed tomography and cadaveric studies tend to report higher values [3,4,12]. In this context, ultrasound (US) has emerged as a sensitive, accessible, and cost-effective tool for detecting accessory ossicles in the foot, demonstrating high diagnostic accuracy for HIO identification [5].

In this study, the prevalence observed using US was notably higher than classical radiographic estimates and aligned with previous US-based investigations [5]. Notably, HIO was bilateral in all identified cases, and no bipartite or multiple ossicles were detected. All ossicles were located intraarticularly in a central position within the plantar capsule, reinforcing current anatomical descriptions of this structure. Yanklowitz et al. using radiographic evaluation found a prevalence of HIO like that observed in our study. This is the only study that reports on the laterality of HIO in the subjects analyzed, as well as its prevalence by sex [13]. The authors established a bilateral prevalence of 94% with a female-to-male ratio of 2.5:1. In the present study, it was not possible to establish the prevalence of HIO by sex, as the sample distribution did not show the homogeneity necessary for studying this variable.

Morphologically, the HIO was visualized on US as a hyperechoic structure with posterior acoustic shadowing, located deep to the interphalangeal joint and separate from the FHL tendon. The dimensions observed in this study were smaller than those reported in cadaveric investigations, likely reflecting the presence of partially ossified structures that are not fully captured in imaging studies.

From a clinical standpoint, HIO has traditionally been regarded as an asymptomatic finding, occasionally associated with os interphalangeus syndrome [14]. However, the absence of symptoms in our cohort suggests that biomechanical alterations may precede clinical manifestations. This distinction is particularly relevant, as it supports the concept of HIO as a potential predisposing factor rather than a direct cause of pathology.

From a biomechanical perspective, the role of HIO remains poorly understood. Previous studies have suggested a potential relationship between HIO presence, HL, and compensatory hyperextension of the interphalangeal joint (IPJ) [9,10]. It has been hypothesized that the presence of the ossicle may alter the mechanical efficiency of the FHL tendon, promoting early dorsiflexion of the proximal phalanx and contributing to altered joint mechanics during gait. The findings of this study indicate that the presence of HIO is associated with increased IPJ extension, supporting its role as a biomechanically active structure rather than a passive anatomical variant. One possible explanation is that the ossicle modifies the mechanical leverage of the FHL tendon by increasing its moment arm, thereby altering force transmission and joint mechanics. This may result in increased flexion torque at the distal phalanx but reduced stability at the MTPJ, promoting compensatory hyperextension at the IPJ [15].

Importantly, this effect was consistent across both open kinetic chain and dynamic conditions, strengthening the hypothesis that HIO has a true functional impact rather than being an artifact of static measurement. The observed relationship between ossicle size and IPJ range of motion, although not strictly linear, further supports a size-dependent biomechanical influence, suggesting that larger ossicles may exert a greater mechanical effect.

In contrast, no significant differences were observed in MTPJ range of motion between groups. This finding suggests that, in a young and asymptomatic population, the presence of HIO primarily affects the IPJ without significantly altering MTPJ function. It is possible that compensatory mechanisms or early-stage adaptations prevent detectable changes at the MTPJ level.

These results support the hypothesis that IPJ hyperextension associated with HIO may precede the development of HL, rather than being solely a compensatory mechanism. Given that the study population consisted of healthy individuals without evidence of HL, the observed biomechanical alterations may represent an early stage in the progression toward more complex forefoot dysfunction.

Some authors have postulated that the presence of a hyperextended or hyperextensible joint is a clinical sign of the existence of HIO and that hyperextension in the absence of a bone occurs in individuals with HL [16]. However, in the present study the discriminative capacity of IPJ extension ROM for HIO presence was moderate, with a sensitivity of 63.2% and limited specificity. While this suggests that increased IPJ extension may be a useful clinical indicator, it should not be considered a definitive diagnostic criterion in isolation. Instead, it may serve as part of a broader clinical and imaging assessment.

Despite these contributions, several limitations must be acknowledged. The use of a young, asymptomatic population limits the extrapolation of findings to clinical populations. The cross-sectional design precludes causal inference, and although US is highly sensitive, it may not fully capture non-ossified structures. Overall, this study provides new insights into the functional role of HIO and supports its consideration as a biomechanically relevant structure rather than a purely incidental finding. Future studies should include longitudinal designs and symptomatic populations to better understand the clinical implications of HIO and its potential role in the development of hallux pathologies.

## 5. Conclusions

The presence of the interphalangeal ossicle of the hallux is associated with increased extension of the IPJ, both in open kinetic chain and dynamic conditions, suggesting a measurable influence on hallux biomechanics. HIO appears to primarily affect IPJ function without significantly altering MTPJ mobility in asymptomatic individuals. The observed increase in IPJ extension may represent an early biomechanical alteration that precedes the development of more complex forefoot disorders, such as HL. Overall, these findings support the clinical relevance of HIO and highlight the need for further longitudinal studies.

## Figures and Tables

**Table 1:** Descriptive statistics of the sample.

	HIO Presence								
	No n = 218 (52.0%)				Yes n = 201 (48.0%)				
	Mean	SD	Median	IQR	Mean	SD	Median	IQR	* p *
Age	30.0	6.8	26	24.0–29.3	27.5	5.3	27	24–29	0.864
Weight	67.3	13.5	66	55–78	67.7	13.4	65	57–78	0.553
Hight	1.68	0.09	1.67	1.62–1.74	1.68	0.09	1.66	1.62–1.73	0.683
BMI	23.6	3.3	23.4	21.3–25.6	23.9	3.7	23.1	21.6–26.0	0.639

SD: Standard deviation; IQR: Interquartile range; *p*: *p*-value.

**Table 2:** Ossicle size measured by US in long and short axis.

	n	Mean	SD	Median	IQR
Long axis 1	201	14.4	4.8	13	11–17
Long axis 2	201	31.0	10.0	30	24–36
Short axis 1	201	18.0	14.7	16	13.0–20.5
Short axis 2	201	46.2	16.4	46	33.0–59.5

**Table 3:** Comparison of ROM in the MTPJ of the hallux in the presence and absence of the HIO.

	HIO										
	No n = 218 (52.0%)					Yes n = 201 (48.0%)					
	n	Mean	SD	Median	IQR	n	Mean	SD	Mean	IQR	* p *
In closed kinetic chain	218	68.8	14.7	69.3	59.0–77.8	201	70.0	14.8	71	61–81	0.204 ^2^
In open- kinetic chain	218	44.7	12.5	45	37.0–52.1	201	44.0	12.3	45.6	35.5–52.0	0.584 ^1^

^1^ *T*-test for independent samples. ^2^ Mann-Whitney U test for independent samples.

**Table 4:** Comparison of ROM in extension of the IPJ of the hallux in the presence and absence of the HIO.

	HIO								
	No n = 218 (52.0%)				Yes n = 201 (48.0%)				
	Mean	SD	Median	IQR	Mean	SD	Median	IQR	* p *
ROM in extension in open kinetic chain	16.2	10.9	15.3	8–23	20.7	11.4	20	12.7–28.0	<0.001 ^1^
ROM in flexion in open kinetic chain	57.9	12.1	58.2	50.0–66.7	56.5	13.6	58	50–65	0.433 ^1^
ROM in extension in closed kinetic chain	13.0	12.4	13	6.0–20.1	17.5	11.0	16.5	11.0–23.7	<0.001 ^1^

^1^ Mann-Whitney U test for independent samples.

## Data Availability

The original contributions presented in this study are included in the article. Further inquiries can be directed to the corresponding author.

## Abbreviations

The following abbreviations are used in this manuscript:

HIO	Hallucal interphalangeal ossicle
HL	Hallux limitus
ROM	Range of movement
MTPJ	Metatarsophalangeal joint
IPJ	Interphalangeal joint
US	Ultrasonography
FHL	Flexor hallucis longus
ICC	Intraclass correlation coefficient
ROC	Receiver operating characteristic
AUC	Area under the curve
OR	Odds ratio
CI	Confidence interval
